# Comparison of Clinical and Radiographic Outcomes According to the Presence or Absence of a Posterior Draw Force during Graft Fixation in Anterior Cruciate Ligament Reconstruction

**DOI:** 10.3390/medicina58121787

**Published:** 2022-12-05

**Authors:** Jin-Ho Cho, Hyun Il Lee, Jae Won Heo, Sung-Sahn Lee

**Affiliations:** Department of Orthopedic Surgery, Ilsan Paik Hospital, Inje University School of Medicine, Goyang-si 10380, Republic of Korea

**Keywords:** anterior cruciate ligament reconstruction, draw, graft

## Abstract

*Background and Objectives:* A reduction forced toward the posterior side during graft fixation may help to lessen anterior tibial translation after ACL reconstruction. The purpose was to compare the clinical and radiological outcomes of graft fixation when a posterior draw was used and when it was not used during anterior cruciate ligament (ACL) reconstruction surgery. *Materials and Methods:* Of 110 patients who had undergone primary arthroscopic ACL reconstruction between January 2017 and August 2020, in all, 76 patients had been operated on without a posterior draw (non-draw group), and 34 patients had received surgery with a posterior draw (draw group). The results of the Lachman test and the pivot-shift test, the Western Ontario and McMaster Universities Osteoarthritis (WOMAC) indexes, the Lysholm scores, the International Knee Documentation Committee (IKDC) subjective scores, and side-to-side difference (STSD) on stress radiography were compared between the two groups. *Results:* The postoperative WOMAC indexes, Lysholm scores, and IKDC subjective scores were similar across both groups. Postoperative STSD (2.4 ± 2.2 for the non-draw group vs. 2.0 ± 2.2 for the draw group; *p* = 0.319) and change in STSD (3.5 ± 3.5 for preoperative STSD vs. 4.3 ± 4.4 for postoperative STSD; *p* = 0.295) were not superior in the draw group. *Conclusions:* The take-home message is that graft fixation with a posterior draw during ACL reconstruction did not result in significantly better postoperative stability. The postoperative clinical outcomes were similar between both groups.

## 1. Introduction

Anterior cruciate ligament (ACL) tears are common in young and active patients. ACL reconstruction is therefore, widely performed, with satisfactory results [1,2,3]. Anatomical ACL reconstruction has allowed us to achieve accurate native ACL positioning and biomechanical characteristics [4,5]. However, certain patients experience poor outcomes with residual instability after surgery [6,7]. Numerous factors are associated with stability after ACL reconstruction. These include graft selection, tunnel position, and extra-articular structure injury (e.g., anterolateral ligament) [8,9,10]. Among these, optimal fixation is one of the most important contributors toward successful reconstruction [11].

**Figure 1:**
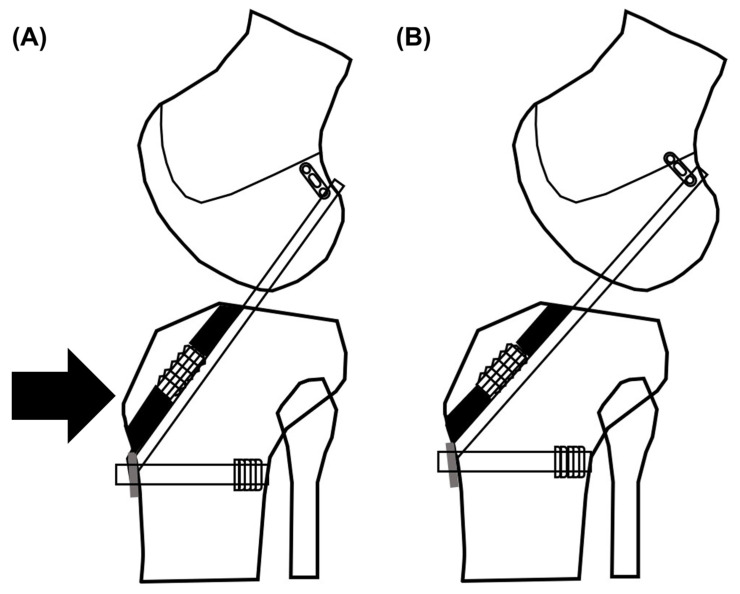
Schematic view of (**A**) a posterior draw force (black arrow) applied and (**B**) a posterior draw force not applied during graft fixation of anterior cruciate ligament reconstruction.

In ACL reconstruction, the optimal fixation of soft tissue grafts, including graft selection, optimal tension, fixative method (aperture fixation vs. suspensory fixation), and knee flexion angle during fixation, remains a controversial topic [12,13,14,15]. Almekinders et al. [16] first reported the concept of static anterior tibial subluxation after ACL injury and the abnormally static relationship between the femur and the tibia with the knee in extension. Subsequently, several studies have investigated static anterior tibial subluxations after ACL injuries [10,17,18,19]. Via cadaveric study, Höher et al. [20] demonstrated that the posterior tibial load during graft fixation restores anterior tibial translation to that of the intact knee. However, to the best of our knowledge, no study has conducted a clinical comparison between graft fixation with posterior load and that without posterior load. Therefore, we considered that reduction forced toward the posterior side during graft fixation could help reduce the anterior tibial translation, as in the case of the cadaveric study mentioned above (Figure 1).

This study aimed to compare the clinical and radiological outcomes of graft fixation with and without a posterior draw. We hypothesized that graft fixation with a posterior draw would reduce the postoperative anterior tibial translation following ACL reconstruction.

## 2. Materials and Methods

### 2.1. Patients

**Figure 2:**
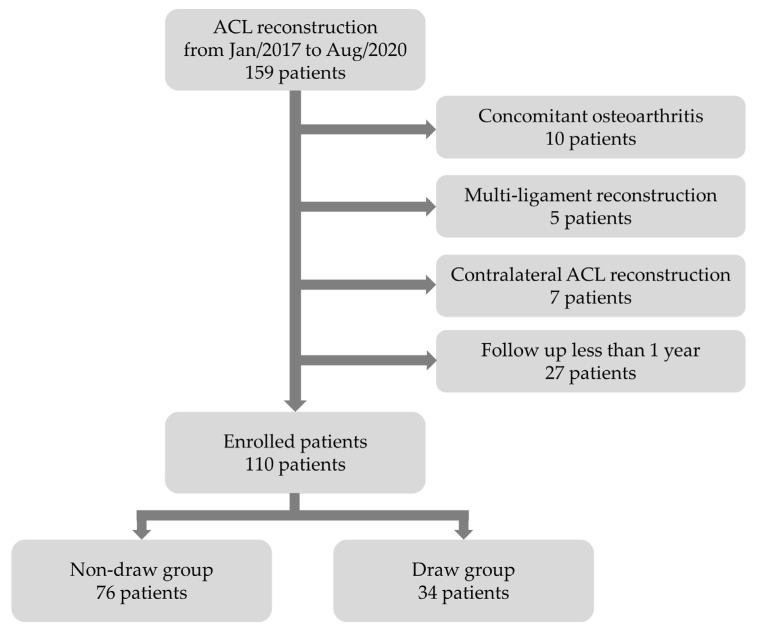
Flow chart describing the patients enrolled in the study.

This was a retrospective study of enrolled patients who had undergone primary arthroscopic ACL reconstruction between January 2017 and August 2020 at our institution. The inclusion criteria were as follows: (1) ACL total rupture, diagnosed by magnetic resonance imaging and arthroscopic examination, (2) ACL reconstruction after diagnosis, (3) age between 17 and 60 years, and (4) a follow-up period of more than 12 months. The exclusion criteria were as follows: (1) osteoarthritic changes in the injured knee, (2) multiple-ligament reconstruction, or (3) contralateral knee ACL reconstruction history [21,22]. A total of 110 patients were enrolled in this study. Patients were divided into two groups: those who had undergone graft fixation with a posterior draw (draw group), and those who had undergone graft fixation without a posterior draw (non-draw group). Of the 110 patients, 76 were in the non-draw group and 34 were in the draw group (Figure 2). The study protocol was approved by our institutional review board, and written informed consent was obtained from all patients (ISPAIK 2021-09-019 at 17 September 2021).

### 2.2. Surgical Procedures

The operations were performed by two fellowship-trained surgeons (J.-H.C. and S.-S.L.). One surgeon preferred the posterior draw of the tibia during graft fixation, while the other did not. The remaining surgical procedures were similar. Each patient was asked to choose the graft type (autograft or allograft) after receiving sufficient information. The hamstring tendon was harvested, and a four-strand double-loop single-bundle graft was inserted in reconstruction with an autograft. If patients opted for an allograft, the allogenous tibialis anterior tendon was used for the ACL reconstruction. A mixed graft was used when the diameter of the harvested autograft was too small for application [23,24].

**Figure 3:**
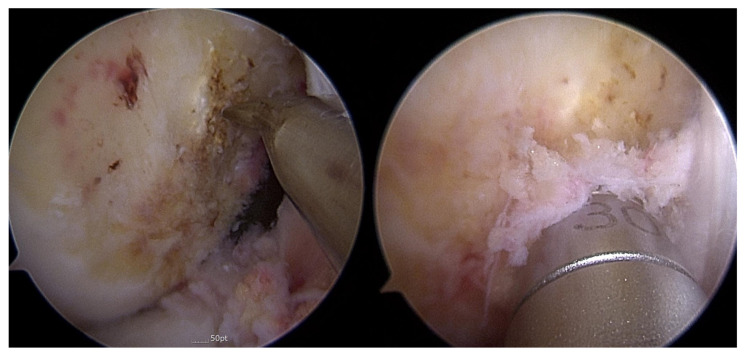
A femoral tunnel was formed using the transanteromedial portal method. A standard anteromedial portal was used for the viewing portal, and a far anteromedial portal was used for the working portal. The center of the anatomical footprint was marked with a microfracture awl. The femoral tunnel was formed using a cannulated reamer.

Portal formation and arthroscopic examinations were performed in the standard manner. Combined meniscal tears were also evaluated. The femoral tunnel was formed using the transanteromedial portal method [25] (Figure 3). The center of the anatomical footprint was marked with a microfracture awl after the removal of the ACL remnant tissue. A 2.4 mm guide pin was inserted with the knee fully flexed, and then a 4.5 mm EndoButton drill (Smith & Nephew, Andover, MA, USA) was used to drill through the far cortex. After assessing what the necessary femoral tunnel length would be, the femoral tunnel was formed using a cannulated reamer.

**Figure 4:**
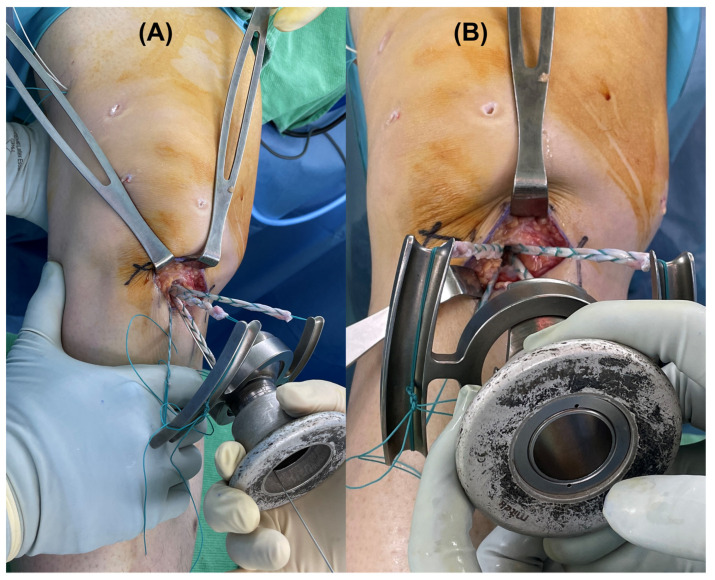
Intraoperative view of (**A**) a posterior draw force applied and (**B**) a posterior draw force not applied during anterior cruciate ligament reconstruction.

To form the tibial tunnel, a guide wire was inserted from the medial tibial cortex into the footprint of the ACL using a Pinn-ACL guide (ConMed Linvatec. Largo, FL, USA), and the tibial tunnel was created using a cannulated reamer. For femoral side graft fixation, the EndoButton (Smith & Nephew, Andover, MA, USA) was used. After the graft was passed, the position of the EndoButton was checked using C-arm fluoroscopy. A tensioner was routinely used to check the initial tension (target: 25 N). For tibial side fixation, a hybrid fixation that combined an intra-tunnel aperture and extracortical suspensory fixation was used [11,12]. A posterior draw force was applied at the proximal tibia when tibial aperture fixation was performed in the draw group, and when no draw force was in the non-draw group (Figure 4).

During the initial four weeks after the reconstruction surgery, crutches for partial-weight-bearing were permitted for walking, and at six weeks, full-weight-bearing walking was permitted. Range-of-motion (ROM) exercises were initiated two days after surgery and reached 120° of knee flexion by four weeks [26]. Straight-leg raises, quadriceps sets, and ankle pump exercises were started on the first day after surgery, and a closed kinetic chain exercise was initiated two weeks postoperatively, and return to sports was allowed after 9 months, depending on the patient’s condition.

### 2.3. Clinical and Radiographic Evaluation

Demographic data, including age, sex, body mass index (BMI), and time from injury to reconstruction surgery, were obtained. Pre- and postoperative clinical outcomes were gathered using the following evaluations: Lachman test, pivot-shift test, Western Ontario and McMaster Universities Osteoarthritis (WOMAC) index [27], Lysholm score [28,29,30], and International Knee Documentation Committee (IKDC) subjective score [31,32]. The Lachman test was graded as 0, 1 (<5 mm), 2 (5 to 10 mm), or 3 (>10 mm) compared to the contralateral knee, and the pivot-shift test was graded as 0 (absent), 1 (glide), 2 (clunk), or 3 (gross) [21,33].

**Figure 5:**
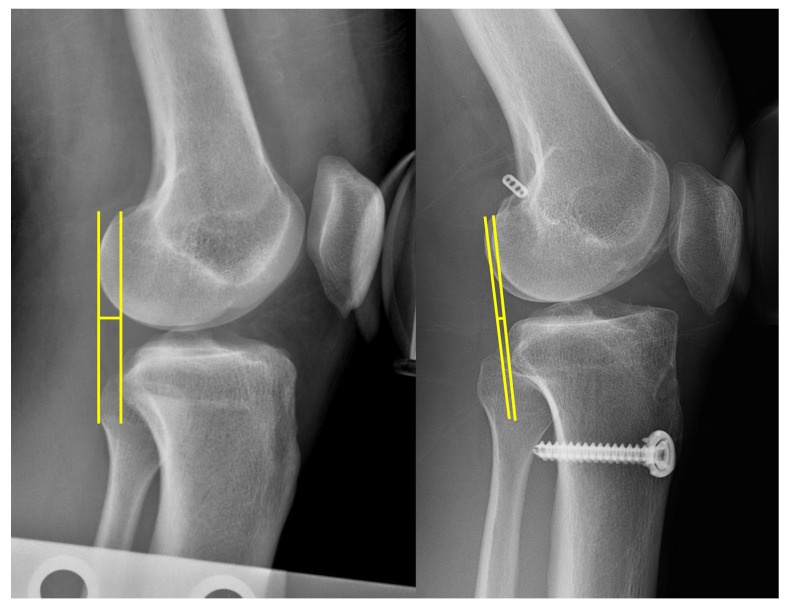
Measurement of preoperative and postoperative anterior tibial translation on anterior stress radiography. (yellow lines) Two perpendicular lines were drawn tangentially to the most posterior contour of the femoral condyle and tibial plateau. Anterior tibial translation was defined as the distance between the two perpendicular lines.

For the quantitative analysis of the anterior tibial translation, stress radiography was used as described previously [34,35,36]. Preoperative and postoperative telos stress radiography (15 kg on the tibia at 20–30° of knee flexion) was evaluated. A pressure plate was placed posteriorly at the mid-calf level, and the other plate was placed at the patella level. A reference line was drawn parallel to the medial tibial plateau joint line. Perpendicular lines from the reference line were drawn tangentially to the most posterior contour of the femoral condyle and the most posterior contour of the tibial plateau. The distance between these two lines was defined as the anterior tibial translation (Figure 5). A side-to-side difference (STSD) was used for the analysis of native laxity. STSD was defined as the difference in the anterior tibial translation between the injured knee and the contralateral non-injured knee. To verify interobserver reliability, the anterior tibial translation was evaluated by two independent orthopedic surgeons (S.H.C. and B.H.K.) specializing in ACL reconstruction, who did not participate in the current study. Intra-observer reliability was checked by having the observers repeat the same measurements six weeks later. Intra-class correlation coefficients (ICCs) were used for inter- and intra-observer reliability assessments.

Preoperative and postoperative outcomes were compared, and all outcomes were compared between the draw and non-draw groups.

### 2.4. Statistical Analysis

To evaluate the normality of the distribution, the Shapiro–Wilk test was used. To compare the clinical and radiographic outcomes between preoperation and postoperation, a paired *t*-test was used for continuous variables, and the Chi-squared test was used for categorical variables. To compare the continuous variables between both groups, Student’s *t*-test or the Mann–Whitney *U* test was used. To compare the categorical variables between both groups, the Chi-squared test was used. Statistical significance was set at *p* < 0.05. All data were analyzed using SPSS version 27.0 (IBM Corp., Armonk, NY, USA). To have a 90% probability of detecting a 1 mm difference in the mean STSD, we needed to enroll 40 patients, assuming an overall standard deviation in 1 mm and a two-tailed alpha-level of 5% [25,37].

## 3. Results

The inter- and intra-observer ICCs of the STSD showed agreement with respect to the reliability of the radiographic measurements (>0.80). Table 1 presents the demographic data.

In all the enrolled patients, the postoperative Lachman test and pivot-shift test grades improved compared to the preoperative grades. Postoperative clinical outcomes, including the WOMAC index, the Lysholm score, and the IKDC subjective score, were greater than the preoperative values. The postoperative STSD was significantly less than the preoperative STSD (2.3 ± 2.2 vs. 6.0 ± 3.4; *p* < 0.001; Table 2).

Demographic data and preoperative outcomes were similar between both groups. The combined meniscal lesions were not statistically different. Postoperative WOMAC indexes, Lysholm scores, and IKDC subjective scores were not greater in the draw group. The superiority of the postoperative STSD (2.4 ± 2.2 for the non-draw group vs. 2.0 ± 2.2 for the draw group; *p* = 0.319) and the change in STSD (3.5 ± 3.5 for preoperative STSD vs. 4.3 ± 4.4 postoperative STSD; *p* = 0.295) were not statistically significant in the draw group (Table 3).

## 4. Discussion

The principal finding of this study is that the posterior draw force during ACL graft fixation did not enhance postoperative stability. Moreover, the postoperative clinical outcomes were similar across both groups.

ACL tears are one of the most common sports injuries. According to a previous study, the annual incidence of ACL tears is 68.6 per 100,000 person-years [1]. The incidence of ACL injury in the general population is higher in males than in females, and the peak in ACL injuries occurs in those aged 19 to 25 years [1,2]. Reconstruction of ACL is still the treatment of choice for a complete ACL tear, and results in excellent postoperative outcomes in more than 90% of the patients [4,5,7,38]. An important goal of ACL reconstruction is to restore knee joint stability. According to a previous study, a non-anatomical tunnel position, injury of the concomitant meniscus, and anterolateral ligament and hamstring tendon graft (compared to a bone–patellar tendon–bone graft) are all associated with inferior postoperative stability following surgery [8,9,10]. Among the numerous variables related to stability, proper fixation is one of the most critical factors. Graft selection, optimal tension, fixative methods, and knee flexion angle during fixation have been investigated previously [12,13,14,15]. Balazs et al. [11] reported that the hybrid methods of tibial-sided graft fixation resulted in a stronger initial fixation and less laxity after healing than an aperture fixation alone or an extracortical suspensory fixation. Yoshiya et al. [12] demonstrated that a set of 25 N during graft fixation showed similar results compared to a set of 50 N. Tanaka et al. reported that the tibial plateau of chronic ACL insufficient patients was positioned more anteriorly [19]. Via cadaveric research, Höher et al. [20] examined anterior tibial translation after ACL graft fixation in different positions. They reported that graft fixation with 30° of knee flexion and posterior tibial load resulted in significantly decreased anterior tibial translation, compared with graft fixation in a neutral position. According to this cadaveric study, we guessed that the reduction forced toward the posterior side might help with respect to graft fixation. However, the postoperative clinical outcomes and stability were similar between both groups in our results. Mae et al. [39] conducted a cadaveric investigation with respect to graft tension during ACL reconstruction. They suggested that the tibia moved proximally and posteriorly during tensioning in the graft fixation stage. We believe that our results support the hypothesis of this study by Mae et al. Adequate graft tensioning causes the tibia to move posteriorly; therefore, an additional posterior draw is not necessary during ACL graft fixation.

Almekinders et al. [16,17,18] first described abnormal tibiofemoral positioning after an ACL injury. They suggested that untreated ACL ruptures result in irreducible anterior tibial subluxation, and that this phenomenon is especially evident on plain radiographs of patients with failed ACL reconstruction. McDonald et al. [10] investigated tibiofemoral subluxation after ACL tears in more detail using magnetic resonance imaging. In their study, patients were divided into four experimental cohorts according to their ACL status: intact ACL, acute ACL disruption (within 2 months of an ACL tear), chronic ACL disruption (more than 12 months after an ACL tear), and failed ACL reconstruction. Their research revealed significantly more medial and lateral compartment subluxation in patients with chronic ACL disruption than in those with normal knees. Patients with acute ACL tears did not, however, show significant subluxation. In the current study, the mean time from injury to surgery was 9.2 and 7.2 weeks in the non-draw and draw groups, respectively. Therefore, it was probably too early for the tibiofemoral subluxation to have occurred. We think there was no significant postoperative STSD difference between the two groups because subluxation had not yet taken place. Further studies are necessary with cohorts that include those with revised ACL reconstructions and chronic ACL deficiencies. These will help to identify the efficacy of a posterior draw force during an ACL graft fixation in patients with tibiofemoral subluxations.

This study has some limitations. First and foremost, two different surgeons performed the two kinds of surgery: ACL reconstruction surgery with a posterior draw and ACL reconstruction surgery without a posterior draw. Although the tunnel-making methods and fixation devices were similar, there could have been some differences in the surgical techniques of the two surgeons. This is a major confounding factor for the interpretation of our results. Second, 76 knees were allocated to the non-draw group, and 34 knees to the draw group. The sample sizes were relatively small and uneven. Follow-up was relatively short and, therefore, the survival analysis or long-term results could not be fully evaluated. Both large-volume and long-term follow-up studies are needed to overcome this limitation in the future. Third, this was a retrospective study, which has inherent limitations and biases. Fourth, ACL tears occurs mainly in young people. However, the age of the patients included in our study varied, which could lead to a bias in the interpretation of the results. Fifth, the posterior draw force was applied manually. Therefore, a constant force would not have been applied to the patients in the draw group, which could lead to a bias in the interpretation of the results.

## 5. Conclusions

Graft fixation with a posterior draw was not shown to be significantly associated with better postoperative stability after ACL reconstruction, as shown by the Lachman test, the pivot-shift test, and STSD on stress radiographs. Postoperative WOMAC indexes, Lysholm scores, and IKDC subjective scores were similar between both groups. Large-volume and long-term follow-up studies are needed in order to clearly identify the effect of a posterior draw on postoperative stability and survival rate.

## Figures and Tables

**Table 1:** Demographic data of all enrolled patients.

No. of patients ultimately enrolled	110
Male:Female	92:18
Age, year	31.3 ± 11.9
Height, cm	171.9 ± 7.1
Weight, kg	75.8 ± 12.6
Body mass index, kg/m^2^	25.5 ± 3.3
Graft	Autograft, hamstring tendon: 66 Allograft, tibialis anterior tendon: 35 Mixed: 9
Time from injury to surgery, weeks	8.5 ± 14.2

Continuous variables are shown as mean ± standard deviation.

**Table 2:** Comparison of preoperative and postoperative outcomes in all patients.

	Preoperative	Postoperative	*p* Value
Lachman test (Grade 0/1/2/3)	1/8/33/68	89/21/0/0	<0.001
Pivot-shift test (Grade 0/1/2/3)	3/20/50/37	99/11/0/0	<0.001
WOMAC index	36.8 ± 23.9	10.6 ± 13.2	<0.001
Lysholm score	54.8 ± 23.9	84.2 ± 13.2	<0.001
IKDC subjective score	49.1 ± 15.2	73.7 ± 13.0	<0.001
STSD, mm	6.0 ± 3.4	2.3 ± 2.2	<0.001

Continuous variables are shown as mean ± standard deviation. STSD, Side-to side difference; WOMAC, Western Ontario and McMaster Universities Osteoarthritis; IKDC, International Knee Documentation Committee.

**Table 3:** Comparison of outcomes between both groups.

	Non-Draw Group	Draw Group	*p* Value
Demographic data			
Number of patients	76	34	
Age, year	30.8 ± 12.1	32.6 ± 11.6	0.472
Sex, male:female	64:12	28:6	0.787
BMI, kg/m^2^	25.2 ± 3.2	26.2 ± 3.6	0.167
Time from injury to surgery, weeks	9.2 ± 14.2	7.2 ± 14.1	0.5
Follow-up period, months	18.6 ± 8.6	16.7 ± 5.4	0.228
Graft (autograft/allograft/mixed)	43/25/8	23/10/1	0.407
Combined medial meniscal tear			
Medial	22 (28.9%)	10 (29.4%)	0.96
Lateral	27 (35.5%)	9 (26.5%)	0.387
Preoperative data			
Lachman test (Grade 0/1/2/3)	0/7/20/49	1/1/13/19	0.499
Pivot-shift test (Grade 0/1/2/3)	3/17/31/25	0/3/19/12	0.217
WOMAC index	35.2 ± 25.9	39.4 ± 18.5	0.396
Lysholm score	55.9 ± 24.5	52.4 ± 21.8	0.472
IKDC subjective score	50.6 ± 15.3	46.1 ± 14.6	0.154
STSD, mm	5.9 ± 3.0	6.3 ± 4.3	0.68
Postoperative data			
Lachman test (Grade 0/1/2/3)	60/16/0/0	29/5/0/0	0.601
Pivot-shift test (Grade 0/1/2/3)	68/8/0/0	31/3/0/0	0.542
WOMAC index	10.3 ± 15.0	10.7 ± 8.1	0.892
Lysholm score	83.5 ± 15.3	86.1 ± 6.5	0.345
IKDC subjective score	74.3 ± 12.4	72.9 ± 14.3	0.603
STSD, mm	2.4 ± 2.2	2.0 ± 2.2	0.319
Change in STSD (pre to post), mm	3.5 ± 3.5	4.3 ± 4.4	0.295

Continuous variables are shown as mean ± standard deviation. STSD, Side-to side difference; WOMAC, Western Ontario and McMaster Universities Osteoarthritis; IKDC, International Knee Documentation Committee.

## Data Availability

Not applicable.
